# Detoxification of Ciprofloxacin in an Anaerobic Bioprocess Supplemented with Magnetic Carbon Nanotubes: Contribution of Adsorption and Biodegradation Mechanisms

**DOI:** 10.3390/ijms22062932

**Published:** 2021-03-13

**Authors:** Ana R. Silva, Ana J. Cavaleiro, O. Salomé G. P. Soares, Cátia S.N. Braga, Andreia F. Salvador, M. Fernando R. Pereira, M. Madalena Alves, Luciana Pereira

**Affiliations:** 1 CEB, Centre of Biological Engineering, University of Minho, 4710-057 Braga, Portugal; ana.rita.silva@ceb.uminho.pt (A.R.S.); acavaleiro@deb.uminho.pt (A.J.C.); catia.braga@ceb.uminho.pt (C.S.N.B.); asalvador@ceb.uminho.pt (A.F.S.); madalena.alves@deb.uminho.pt (M.M.A.); 2 Laboratory of Separation and Reaction Engineering, Laboratory of Catalysis and Materials (LSRE-LCM), Faculty of Engineering, University of Porto, 4200-465 Porto, Portugal; salome.soares@fe.up.pt (O.S.G.P.S.); fpereira@fe.up.pt (M.F.R.P.)

**Keywords:** anaerobic reduction, adsorption, ciprofloxacin, magnetic carbon nanotubes, redox mediators, toxicity

## Abstract

In anaerobic bioreactors, the electrons produced during the oxidation of organic matter can potentially be used for the biological reduction of pharmaceuticals in wastewaters. Common electron transfer limitations benefit from the acceleration of reactions through utilization of redox mediators (RM). This work explores the potential of carbon nanomaterials (CNM) as RM on the anaerobic removal of ciprofloxacin (CIP). Pristine and tailored carbon nanotubes (CNT) were first tested for chemical reduction of CIP, and pristine CNT was found as the best material, so it was further utilized in biological anaerobic assays with anaerobic granular sludge (GS). In addition, magnetic CNT were prepared and also tested in biological assays, as they are easier to be recovered and reused. In biological tests with CNM, approximately 99% CIP removal was achieved, and the reaction rates increased ≈1.5-fold relatively to the control without CNM. In these experiments, CIP adsorption onto GS and CNM was above 90%. Despite, after applying three successive cycles of CIP addition, the catalytic properties of magnetic CNT were maintained while adsorption decreased to 29 ± 3.2%, as the result of CNM overload by CIP. The results suggest the combined occurrence of different mechanisms for CIP removal: adsorption on GS and/or CNM, and biological reduction or oxidation, which can be accelerated by the presence of CNM. After biological treatment with CNM, toxicity towards *Vibrio fischeri* was evaluated, resulting in ≈ 46% detoxification of CIP solution, showing the advantages of combining biological treatment with CNM for CIP removal.

## 1. Introduction

Pharmaceuticals are considered emergent micropollutants by the European Commission due to their potential environmental, ecotoxicological, and sociological risk [1]. A wide range of pharmaceuticals, such as antibiotics, anti-inflammatory drugs, anxiolytics and hormones, are not totally metabolized by humans and animals, being excreted to the environment. Hospital and pharmaceutical industries, as well as domestic wastewater, are potential sources of contamination [2]. In wastewater treatment plants (WWTP), these compounds are not completely removed nor mineralized, and end up in natural water bodies or in soils, as well as in drinking waters, and biomagnify in food chains [2,3,4,5]. Furthermore, in nature, continuous contact between bacteria and such substances increases the number of multi-drug resistant bacteria [6], with negative consequences for human health.

The most prescribed pharmaceuticals coincide with the ones detected in the WWTP [7,8], as, e.g., ciprofloxacin (CIP), a broad-spectrum fluoroquinolone antibiotic, that is extensively used for the treatment of bacterial infections in humans and animals. Up to 72% of the dosed CIP may exit the target organism in unaltered form, thus reaching the WWTP and the environment [3,4]. Fluorinated antibiotics are generally present in WWTP at low concentrations, ranging from nanograms to micrograms per liter. For CIP, concentrations up to 31 mg L^−1^ (i.e., 0.094 mmol L^−1^) were detected in the effluent of WWTP treating wastewater from several pharmaceutical industries [9]. The presence of CIP in effluents from WWTP reveals the inefficiency of the implemented processes for treating wastewater containing this antibiotic. Nevertheless, significant amount of CIP is retained in the WWTP by adsorption on the sludge [4,10,11]. Indeed, despite the low K_ow_ value of CIP (i.e., 0.28 [12]), Lindberg et al. [13] showed that more than 70% of the CIP entering a conventional WWTP in Sweden was removed by sorption, and was concentrated in the digested sludge. Digested sewage sludge is commonly added to agricultural soil as fertilizer, what may contribute to disseminating CIP in the environment and facilitate its entrance in the food chains.

Although removal of pharmaceutics in WWTP is attributed mostly to sorption on sludge [4,14], biodegradation also occurs [10]. The aerobic bacteria *Labrys portucalensis* is able to degrade CIP in pure culture, when supplemented with an additional and easily biodegradable substrate [3]. Furthermore, CIP was shown to be used as sole carbon source by a complex microbial community retrieved from a drinking water biofilter [15]. Although biodegradable by microbial communities, CIP was found to be toxic to some microorganisms. For example, in anaerobic sludge, acetoclastic methanogens showed higher sensitivity to the presence of CIP, while hydrogenotrophic methanogens indicated low susceptibility to this compound [16]. In another study, anaerobic microbial communities were found to tolerate CIP concentrations up to 50 mg L^−1^ (0.15 mmol L^−1^) [17]. The vast physiological diversity of anaerobes is still an open field to explore for the development of novel biotechnological processes. In fact, anaerobic biodegradation of pharmaceuticals seems promising, but it is still poorly explored and little is known about the mechanisms involved. The analgesic acetylsalicylic acid [18], the anti-inflammatories ibuprofen and diclofenac, the beta-blocker metoprolol [19], and the antibiotics benzylpenicillin [20], tetracycline [21], norfloxacin [22], sulfamethoxazole, and trimethoprim [23], were found to be degraded anaerobically, with the last two antibiotics achieving removal efficiencies above 84%. Additionally, in anaerobic bioreactors, the electrons produced during the oxidation of organic matter can potentially be used for the biological reduction of pharmaceuticals, which may represent an alternative way of promoting pharmaceuticals biotransformation. Notwithstanding, the low transformation rates of many recalcitrant compounds in anaerobic bioprocesses represent a drawback to their application [24]. These low rates are mainly due to electron transfer limitations, that may be overcome by the application of redox mediators (RM).

RM are organic molecules that can reversibly be oxidized and reduced, acting as an electron carrier in multiple redox reactions. RM can accelerate the global reaction rates, by lowering the corresponding activation energy [25]. The reduction rates of dyes and aromatic amines were greatly improved, in batch and in continuous anaerobic bioreactors, by adding low amounts of different carbon nanomaterials (CNM) and magnetic nanomaterials (MNM) as RM [26,27,28,29,30]. In some cases, no reduction occurred in the absence of the tested nanomaterials [26,27,28,29,30]. CNM efficiency as RM is mainly due to their high surface area, proper pore size and excellent catalytic properties [25,31,32]. In addition, insoluble materials like CNM can be retained in the bioreactors, avoiding the need of continuous supplementation during the process [27]. In order to facilitate the recovery of these materials from bioreactors, which can then be further reutilized, magnetic composites may be used instead. Magnetic composites—i.e., core(ferrite, FeO)-shell (carbon, C) composites and carbon nanotubes (CNT) impregnated with 2% of Fe (CNT@2%Fe) were proved as very efficient RM in the anaerobic reduction of the recalcitrant azo dye Acid Orange 10 (AO10), where a 76-fold increase of the AO10 reduction rate was obtained with CNT@2%Fe [30]. The magnetic properties of those composites allowed their recovery from the reactors, by using a magnetic field, and enabled their reuse in successive cycles, maintaining the RM characteristic [30,33,34].

The anaerobic removal of pharmaceuticals assisted by nanomaterials thus appear as a promising strategy that deserves to be investigated. In this study, commercial and tailored CNT were evaluated as RM in the anaerobic removal of CIP. Tailored CNT were prepared from the commercial CNT though a set of surface modifications, aiming to obtain materials with different surface chemical groups (acidic and basic) while maintaining their main textural properties. Pristine and tailored CNT were characterized, and first utilized in chemical reduction tests, to evaluate the performance of the CNM as RM in the chemical reduction of CIP. The best CNM was further tested in biological anaerobic assays. Magnetic CNT were also prepared, by incorporation of iron (CNT@2%Fe), characterized and used in the biological experiments, considering that these materials are easier to recover and may be reused, which is important for applied biological treatment processes. The potential contribution of adsorption and biodegradation processes was assessed. Detoxification of CIP solutions was evaluated towards *Vibrio fischeri*, before and after the treatment.

## 2. Results and Discussion

### 2.1. Textural and Chemical Characterization of CNM

**Figure 1:**
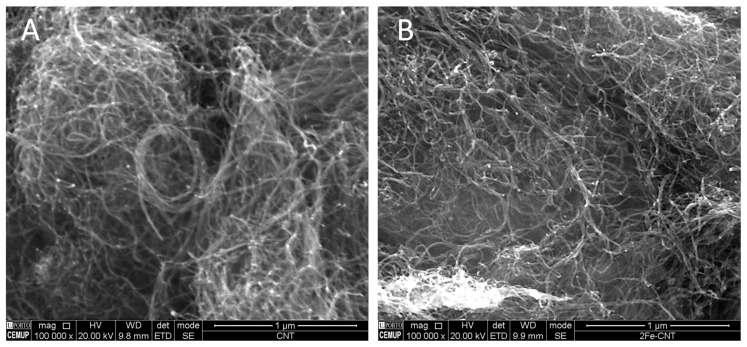

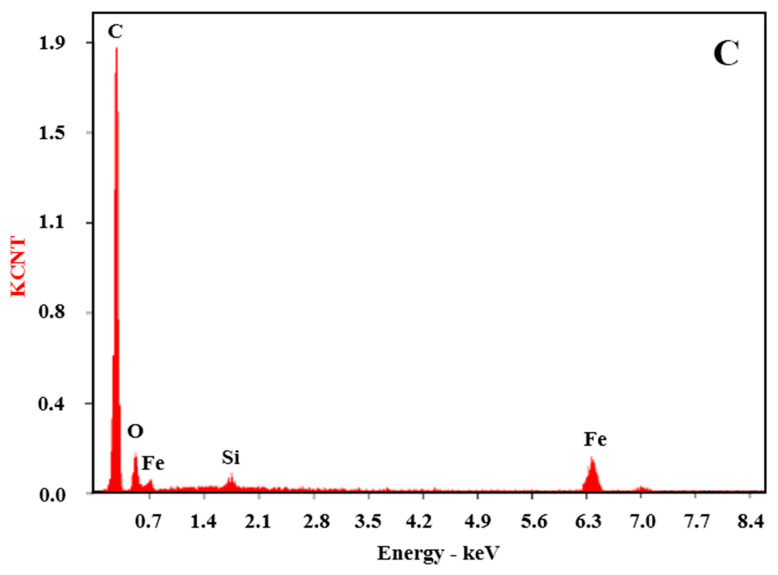
Scanning Electron Microscopy (SEM) images of (**A**) carbon nanotubes (CNT) and (**B**) CNT@2%Fe and (**C**) Energy Dispersive Spectroscopy (EDS) analysis of CNT@2%Fe sample.

The results of the textural and elemental analysis of the different CNM are presented in Table 1. CNT are mesoporous nanomaterials, presenting a specific surface area (*S*_BET_) of 201 m^2^ g^−1^ and pore volume (*Vp*) of 0.416 cm^3^ g^−1^. The functionalization procedures applied promoted a slight increase of these two parameters, both in CNT_N and in CNT_HNO_3_ (Table 1), showing the occurrence of changes on the CNT structure. The oxidative treatment may cause breaks on the walls and open up of the tips of the nanomaterial, leading to a slight increase of the *S*_BET_ [35]. On the other hand, the CNT_N samples were submitted to a ball milling, which promotes a better dispersion of the CNT in the first stage of the process, and leads to shorter CNT by breaking up the tubes without affecting their diameter [36]. Previous CNM characterization by TEM [37], revealed that pristine CNT structure consisted in aggregates of tubes highly entangled, curved, and twisted with each other, and that the ball-milling (sample CNT_N) reduced significantly this entanglement because the mechanical treatment breaks up the tubes, shortening the CNT, and increasing the surface area (Figure S1; Table 1). Thus, the functionalization procedures applied improved the accessibility of the nanotubes, and the increasing of *S*_BET_ on the CNT’s disentangling could be associated with the increase of the *Vp*. On the other hand, despite the CNT impregnated with 2% of iron demonstrating a slight decrease in the surface area, this decrease is not considered significant since the iron quantity introduced in the carbon network is low, being the surface area of these CNM maintained similar to the original CNT (Figure 1). Scanning Electron Microscopy with Energy Dispersive Spectroscopy (SEM/EDS) analysis of CNT and CNT@2%Fe samples confirms that these CNM are tubes highly entangled and twisted, and that the impregnation of Fe on CNT structure was successful (Figure 1C).

The surface chemistry of the CNT was also modified by the applied treatments, consequently promoting changes on the surface charge of the nanomaterials [37], as assessed by the pH_PZC,_ of the CNM, since the pH_PZC_ is related with the surface groups present in the materials surface. The oxidative treatment caused a decrease in the pH_PZC_ from 6.6 (pristine CNT) to 2.2 (CNT_HNO_3_) (Table 1), due to the incorporation of a large amount of oxygen containing groups [26,38,39,40]. The introduction of nitrogen functionalities on the CNT_N by the milling process with melamine followed by thermal treatment only slightly increases the pH_PZC_ when comparing to the original CNT [27]. In addition, it is not expected that the impregnation of Fe in CNT causes changes in the pH_PZC_ of pristine CNT, which was confirmed by the experimental determination of CNT@2%Fe pH_PZC,_ that was 6.5 (Table 1).

From the elemental analysis (Table 1), it is possible to observe that all CNM are mainly composed of carbon. Pristine CNT presented a very low percentage of hydrogen and oxygen, while CNT_HNO_3_ demonstrated a higher amount of oxygen (1.25%), proving the presence of oxygen-rich groups in this sample. Moreover, the incorporation of N-groups on CNT_N was successful, with 1.69% of N being present in this sample.

### 2.2. Effect of CNM on the Chemical Reduction of CIP

The results of the chemical reduction of CIP by sulfide in the absence of oxygen and at pH 7.0, are presented in Table 2. No reduction could be detected in the assays with CNT_HNO_3_ or in the absence of CNM, revealing the recalcitrant nature of this compound [4,41]. Despite that, in the presence of pristine CNT, 42.6 ± 5.0% of the added CIP was removed, at a reaction rate of 0.082 ± 0.001 mmol L^−1^ d^−1^. CIP was also removed in the assays with CNT_N, although at a lower extent and rate (i.e., 30.1 ± 8.6% at 0.063 ± 0.001 mmol L^−1^ d^−1^, respectively), showing the pertinence of screening tailored materials for specific applications, in this case for CIP reduction Indeed, a previous work, on the chemical reduction of azo dyes by Na_2_S under anaerobic conditions at different pH values (5, 7 or 9), in the presence of pristine and tailored (oxidized or thermal treated) activated carbon (AC) revealed that the pH_PZC_ of the materials, and also the charge of the dyes, played an important role in the reduction efficiency [26]. In that work, thermal treated AC had better efficacy comparatively to pristine and oxidized AC. The same behavior was then proved in the biological experiments for the reduction of the same dyes [26].

One of the explanation of why the effects of CNM differ according to the pollutant in question and conditions of the process, is related with their amphoteric character, i.e., their surfaces may become positively or negatively charged, depending on their pH_PZC_ and on the pH of the solution. The CNM surface becomes negatively charged at pH > pH_PZC_ and positively charged at pH < pH_PZC_ [30,37].

CIP has amphoteric character as well, due to the bicyclic aromatic ring skeleton with a carboxylic acid group (C-3, pK_a1_ of 5.90 ± 0.15), a keto group, and a basic amino moiety in the piperazine ring (C-7, pK_a2_ of 8.89 ± 0.11). So, depending on the pH conditions, CIP can be in different ionic forms, showing different physicochemical (e.g., on solubility and lipophilicity) and biological behavior [5,42,43]. At pH below 5.90, CIP is in the cationic form (CIP^+^) due to the protonation of the amine group in the piperazine moiety, and, at pH above 8.89, it is in the anionic form (CIP^−^), because the carboxylic group lacks a proton. In the range between 5.90 and 8.89, the balance of the two groups stabilizes CIP, which acquires the neutral zwitterionic form (CIP^±^) [44,45]. Among the three ionic species of CIP, CIP^±^ is the most hydrophobic one, owing to the lowest solubility at the neutral pH [42,46,47]. As the assay was conducted at pH 7.3 ± 0.2, CIP^±^ was predominant, and hydrophobic may prevail over electrostatic interactions. Therefore, the main mechanisms proposed are the hydrophobic, hydrogen bond, electrostatic, and/or π-π electron donor–acceptor interactions [48,49,50].

By knowing the pH_PZC_ of the tailored CNM, it is possible to predict the interaction between the CNM and CIP. At pH 7, CNT (pH_PZC_ 6.6) and CNT_N (pH_PZC_ 6.7) possess pH_pzc_ closer to the neutrality, while CIP is in its neutral zwitterionic form. Thus, the electrostatic interaction between these CNM and CIP^±^ may be unfavourable, but hydrophobic interactions are enhanced, which may explain the removal of CIP with these two materials (Table 2). Among them, and contrarily to previous results that indicated the best efficiency of the CNT when doped with N [37], in this work the pristine CNT was shown as the best RM regarding CIP chemical reduction. CNT and CNT_N used in this study have similar pH_PZC_, but the presence of N group on the surface of tailored CNT seems to hinder the CIP accessibility to the carbon network, and, consequently, its removal from the solution, since the N groups may fill the empty spaces of the carbon structure interfering with the adsorption of large molecules [35,51]. The low adsorption on the material may also decrease the electron transfer and consequently, the reduction of CIP. On the other hand, CNT_HNO_3_ possess negative charge at the medium pH 7 and a decreasing tendency to dispersive interactions, revealing some repulsive interactions with CIP, which may justify the lack of CIP reduction under this condition. Similarly, oxidative treatment with HNO_3_ worsen the catalytic efficiency of AC as RM in the chemical reduction of the dyes [26].

Adsorption of CIP on nanomaterials was also expected as CNM have been shown as good adsorbents for organic and inorganic compounds, due to their high specific surface area [26,35]. The contribution of the adsorption phenomenon was evaluated in the absence of Na_2_S and accounted for circa 3% of CIP removal for all the materials, after reaching the adsorption–desorption equilibrium (Table S1).

Previously, it was stated that higher *S*_BET_ promotes greater removals of organic and inorganic molecules [26,27,37]. However, *S*_BET_ is not the only parameter involved in the removal mechanisms, and in this study, despite lower *S*_BET_ of CNT comparatively to CNT_N, it was more effective on promoting CIP removal, demonstrating the strong influence of the CNM surface chemistry. Based on this observation, CNT were chosen as RM in CIP biological removal experiments.

### 2.3. Biological Removal of CIP Assisted by CNM under Anaerobic Conditions

#### 2.3.1. CIP Removal under Anaerobic Conditions

**Figure 2:**
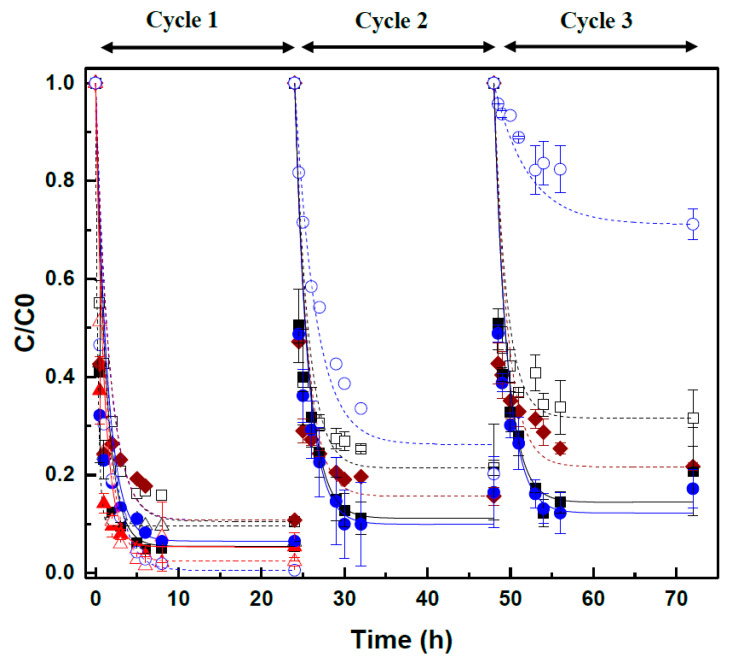
First-order rate curves of CIP removal in the anaerobic assays, performed in the absence of CNM (GS+CIP+E) (■), with CNT (GS+CIP+E+CNT, 1st cycle only) (▲) and in the presence of CNT@2%Fe (GS+CIP+E+CNT@2%Fe) (●). Blank controls (without ethanol) are also shown: without CNM (GS+CIP) (□), with CNT (GS+CIP+CNT, 1st cycle only) (Δ) and with CNT@2%Fe (GS+CIP+CNT@2%Fe) (♦). Abiotic controls (without granular sludge (GS)) in the presence of CNT (CIP+E+CNT, 1st cycle only) (Δ) and CNT@2%Fe (CIP+E+CNT@2%Fe) (○) are presented as well.

The concentration of CIP in the bulk media decreased in the incubations performed with granular sludge (GS), ethanol, CNT, or CNT@2%Fe, but also in the control assays, including abiotic controls, although at a lesser extent (Figure 2). The reactions followed the first-order kinetics and the calculated removal extents and rates are shown in Table 3. In the blank assays (without ethanol) performed in the absence of CNM (GS+CIP), the percentage of CIP removal was 90 ± 0.1% at a reaction rate of 1.16 ± 0.1 d^−1^, which suggests a high adsorption of CIP on the anaerobic sludge. However, when ethanol was added as substrate (GS+CIP+E), CIP reduction increased to 95 ± 1.0%, and occurred at the reaction rate of 1.67 ± 0.4 d^−1^. This improvement pinpoint to the contribution of biological activity in CIP removal. Indeed, the anaerobic sludge consumed ethanol, and the formation of acetate and methane (CH_4_) was verified, as it will be further discussed. The rate of biological removal of CIP was upgraded in the presence of CNM: 1.34-fold higher with CNT and 1.53-fold higher with CNT@2%Fe, resulting in removals of 97 ± 0.7% and 94 ± 0.5%, respectively. This increment suggests stimulation of the biological activity by CNM, so acting as RM on the reductive reactions [30]. In a previous work [27,30], the improvement of the extent and rate of the biological reduction of AO10 obtained with CNT was explained by the CNT’s high pore volume and also by the high content of active sites (electron π rich sites on their basal planes), as well as the low concentration of electron-withdrawing groups, which favor the electron transfer and therefore, the reduction of the compounds. Due to the fluorine group present in the molecular structure, CIP is a strong π-acceptor compound [52].

Notwithstanding, the removal of CIP in abiotic controls (without GS) was 98 ± 0.5%, for CNT, and 99 ± 0.4%, for CNT@2%Fe (Table 3), and was likely due to the contribution of CIP adsorption onto the nanomaterials [42,46,47]. Likewise, previous results with an azo dye have also shown that the presence of iron on CNT@2%Fe contributed to enhance the reduction capacity under abiotic conditions, which was attributed to the transfer of electrons first from nanoscale iron to carbon, and finally to the dye [30]. The same process may have occurred in the abiotic assays performed in the presence of this CNM (i.e., CIP + E + CNT@2%Fe), and could have also potentially contributed for the removal of CIP verified both in the biological assays (GS + CIP + E + CNT@2%Fe) and in the blanks (GS + CIP + CNT@2%Fe) containing this magnetic CNT.

The results of the first 24 h suggest the combined contribution of adsorption and biological activity in the removal of CIP. However, regarding the slight differences observed in this first cycle between the blank, abiotic, and biological assays, it was difficult to distinguish between the different phenomena contributing for CIP removal, because in this cycle adsorption may be the main mechanism, once biomass and CNT are not yet saturated (Figure 2).

In this sense, considering the higher and faster CIP removal achieved in the assays with CNT@2%Fe, and taking into account that these CNM have magnetic properties which favor their recovery and reuse, two additional cycles of 24 h were performed with this material, as well as the blank and abiotic controls (Table 3). The aim was to provide clear evidence on the role of biological degradation in CIP removal, since GS and CNM saturation is expected to occur over the cycles, thus decreasing the contribution of the adsorption phenomenon. At the same time, the reusability and the evolution of the catalytic properties of CNT@2%Fe could be evaluated.

Indeed, in the second and third cycles, lower extents of CIP removal were obtained in all the assays, comparing to the first cycle (Figure 2; Table 3), possibly due to saturation of the adsorbent materials. This decrease was more pronounced in the abiotic assays (CIP + E + CNT@2%Fe), where CIP removal reached only 29 ± 3.2% at the end of the third cycle. In the biological assays with ethanol, a high CIP removal capacity was still verified in the third cycle, both in the presence and absence of CNT@2%Fe (i.e., 88 ± 4.1% and 86 ± 2.2%, respectively), highlighting the importance of the biological activity in this process. In these assays, microorganisms may be oxidizing ethanol and reducing CIP, which acted as final electron acceptor.

**Figure 3:**
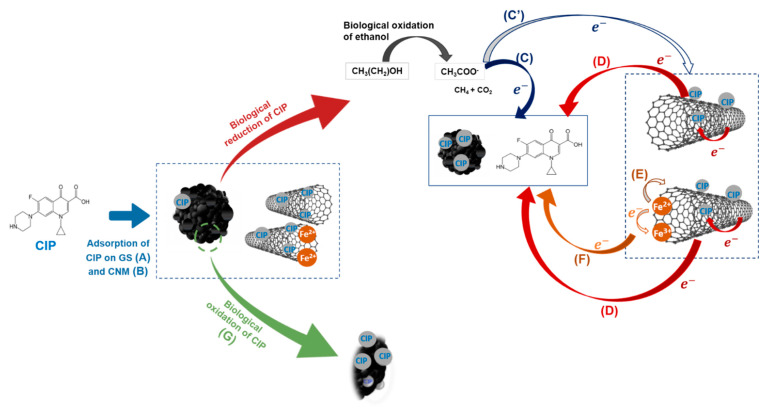
Proposed mechanisms of CIP removal: adsorption of CIP on GS (A) and on CNT or on CNT@2%Fe (B); biological reduction due to electron (e^−^) flow from the biological oxidation of ethanol to CNM (C’) or CIP (in solution and/or adsorbed on sludge) (C); biological reduction in the presence of CNM (D) due to e^−^ transfer from the oxidation of ethanol to CNM and then to CIP (in solution or adsorbed on sludge or on CNM), and due to e^−^ transfer to CNT from the abiotic oxidation of Fe^2+^ (E) and then to CIP (adsorbed or in solution) (F). Further, oxidation of CIP (in solution and adsorbed on sludge) by the anaerobic microorganisms may occur (G). All these mechanisms may occur independently or combined.

The second and third cycles, make clear the contribution of the several removal mechanisms, including adsorption and degradation, occurring simultaneously in the system, but biological reactions might be preponderant in those two last cycles owning the saturation of GS and CNT@2%Fe (Figure 3) [10,53]. In the blank assays without ethanol (GS + CIP and GS + CNT@2%Fe), and after three cycles, 78 ± 0.8% and 68 ± 5.7% of the added CIP was removed in the presence and absence of the CNM, respectively. These values are higher than in the abiotic assay, showing that besides CIP adsorption on CNM and GS, biological removal also occurs in the blank assays, without ethanol as electron donor. This can be justified by the utilization by anaerobic microbial community of other electron donors originated from dead microbial cells, metabolites excreted during cell decay. Alternatively, microbial oxidation of CIP can be hypothesized. As sole carbon source, CIP has only been oxidized in the presence of sulfate or nitrate, and CIP oxidation in the absence of any external electron acceptor other than bicarbonate (i.e., in conditions similar to the ones in this study) was never reported [54].

The catalytic properties of CNT@2%Fe were maintained over the cycles, as shown by the higher removal extent and reaction rates verified in the biological assays (GS+CIP+E+CNT@2%Fe), both in the second and third cycles, comparatively to the assay in its absence (GS+CIP+E) (Figure 2; Table 3). Despite the statistically similar reaction rates obtained in the presence and absence of CNT@2%Fe, in the second and third cycles, probably as a result of the adaptation of the microbial community to the substrate and to CIP [55], the presence of CNM could be determinant in the initial stage of the reaction, speeding up the reaction rates and improving the reductive system.

#### 2.3.2. Assessment of the Biological Activity during CIP Removal

The activity of the anaerobic microbial community was assessed in the biological assays by measuring the decrease in ethanol concentrations along the time, coupled to acetate and methane (CH_4_) production (Table 4, Figure S2 and Table S2). Ethanol was totally consumed by the anaerobic granular sludge, both in the presence and in the absence of CNM, in all the cycles (Table S2 and Figure S2). The maximum methane concentration produced in all the conditions is in agreement with the value that could be expected from the stoichiometric conversion of ethanol to methane (i.e., 45 mmol L^−1^ CH_4_ from 30 mmol L^−1^ ethanol, Equations (1)–(3), Table S2).

(1) $$C_{2}H_{5}O^{-}+H_{2}O\;\to \;CH_{3}COO^{-}+2H_{2}\;(\mathrm{acetogenesis})\;$$

(2) $$CH_{3}COO^{-}+H_{2}O\;\to CH_{4}+HCO3^{-}(\mathrm{methanogenesis})$$

(3) H2+14HCO3−+ 14H+→14CH4+34H2O (methanogenesis)

Ethanol is converted initially to acetate and H_2_ (acetogenesis), and acetate and H_2_ are further converted to CH_4_ (methanogenesis) [56,57]. The monitoring of acetate concentration over the time in the assay GS+CIP+E+CNT@2%Fe showed a transient accumulation of this compound in the medium, being then almost completely consumed until the end of the cycles (Figure S2). In all the conditions tested, acetate was present at low concentrations (<5 mmol L^−1^) at the end of each cycle, demonstrating the total conversion of the substrates to CH_4_. Indeed, both acetoclastic and hydrogenotrophic methanogens, which perform acetate and hydrogen conversion to methane, respectively, were detected in the inoculum sludge utilized in these experiments. Acetate conversion was probably carried out by *Methanosaeta* species as it was the only acetoclastic genus detected, and in a high relative abundance (~20%) (Table S3). The transient accumulation of acetate may be due to the adaptation of *Methanosaeta* species to the incubation conditions, i.e., the presence of carbon nanomaterials and/or the presence of CIP. On the other hand, different hydrogenotrophic methanogens could be converting hydrogen to methane as several species could be detected in the inoculum sludge, namely, *Methanobacterium* and *Methanolinea* in relative abundances of 9%, and *Methanospirillum* and *Methanobrevibacter* which were less abundant (0.07% and 0.003% relative abundance, respectively). The bacterial community was much more diverse and therefore, it is not possible to confidently infer on the function of specific microorganisms in the assays. Nevertheless, *Geobacter* species were detected in high abundance (over 14%) and once these microorganisms are known as ethanol degraders, they might have had a relevant contribution in the conversion of ethanol to acetate and hydrogen in these experiments.

The presence of CIP did not inhibit the methanogenic activity (Figure S2; Table 4), since there were no significant differences in the CH_4_ production rate from ethanol when the sludge was incubated in the presence and absence of CIP (GS+CIP+E and GS+E), in each cycle. These results are in agreement with the ones previously observed by Silva et al. [16].

The consumption rate of ethanol, and the production rate of CH_4_, increased at each cycle, probably as a result of the microbial community growth, resulting in higher ethanol conversion rates (Table 4).

#### 2.3.3. Mechanisms of CIP Removal

The obtained results, taken all together, point to the occurrence of different mechanisms of CIP removal, namely adsorption on sludge and/or on CNM, and biological removal by oxidation and/or reduction, which are accelerated by the presence of CNM (Figure 3). The results suggest that adsorption phenomena likely occurred in the beginning until saturation of GS and CNM, and biological reactions prevailed after reaching the adsorption/desorption balance. In fact, in the biological assay with ethanol and without CNM, adsorption of CIP on GS and its reduction due to electrons generated by the oxidation of ethanol, or biological oxidation of CIP, possibly occurred (Figure 3). The event of biological reduction of CIP by the electrons generated from the oxidation of ethanol (Figure 3C,C’) probably explain the higher percentage of removal as compared with the assay without substrate where only adsorption (A) and biological oxidation may occur (Figure 3G). When CNM are present, besides absorption of CIP on GS and on the nanomaterials (Figure 3A,B), respectively), improvement of the reaction rates by the CNM, which act as electron shuttles, may justify the high extent of removal (Figure 3C’–D).

In addition, the high rates obtained with the CNT@2%Fe in the biotic and abiotic condition, may result from the fact that besides CIP adsorption and reduction by electrons generated from ethanol oxidation, electrons may flow from Fe^2+^ to CNT and then to CIP (adsorbed on CNT@2%Fe and on GS, and free in solution), as represented in Figure 3E,F [30]. It is important to note that a dynamic adsorption/desorption process to GS and nanomaterials is probably occurring during the incubation period. Adsorption phenomena are required for the success of biological degradation of micropollutants, since the flow of electrons is favored by the proximity between the microorganisms, the catalyst and the pollutant [30,58]. On the other hand, Salvador et al. [58] observed a good binding of CNM on anaerobic microorganisms, which resulted in the improvement of microbial activity.

Adsorption on GS was expected based on previous studies reporting that the removal of CIP is mainly due to adsorption on activated sludge and CNM, rather than biodegradation [13,59,60,61]. The adsorption of CIP onto the sludge is a spontaneous, exothermic and a linear process that includes both physisorption and chemisorption [10,11]. As mentioned above, at neutral pH, CIP mainly presents zwitterionic form (CIP^±^) with –NH^2+^ and –COO^−^ groups [49,62,63]. The functional groups present on anaerobic sludge, such as C–O, C–O–C, N–H, O–H and COOH provide binding sites for CIP^±^ adsorption [10]. These functional groups act as strong electron acceptors and conjugated with the π electron-donating groups of CIP (N–H and O–H) form a π-π electron donor–acceptor system [10]. On the other hand, the O–H groups present on the sludge can be conjugated with the COOH and C=O groups of CIP and the COOH and N–H groups on sludge surface may also form hydrogen bonds with O–H group in CIP molecule [10]. Additionally, the negative surface charge of sludge at neutral pH could also stimulate the CIP^±^ adsorption onto the sludge via electrostatic attraction and cation exchange [11]. Thus, the high CIP adsorption onto the sludge could be attributed to the multiple adsorption mechanisms, including hydrophobic interaction, electrostatic attraction, cation exchange and bridging, π-π interaction, and hydrogen bond effect [64].

Furthermore, the adsorption of CIP on CNT is spontaneous when Gibbs free energy (ΔG^0^) is negative [42,65,66], where the binding mechanisms mainly associated to this phenomenon is physisorption [42,49,67].

On the other hand, the sorption energy decreased with the increasing of CIP loading, hence, CIP molecules first occupied the high-energy sorption sites at low concentration and then spread to low-energy sorption sites [42,68]. Furthermore, recent studies have reported that the removal of pharmaceuticals by anaerobic sludge occurs initially by sorption, but after the equilibrium being reached, the mass-transfer driving force no longer affects the pharmaceutical uptake due to the absence of a concentration gradient. Being the biodegradation mechanism the major removal route in the system [10,53,68,69,70,71,72].

### 2.4. Toxicity Assessment with Vibrio fischeri

Evaluation of the toxicity of the samples was performed after the biological anaerobic treatment proposed, to assess the detoxification extent (Table 5). The initial CIP solution (0.015 mmol L^−1^) led to an inhibition of 56 ± 10% of the luminescence of *V. fischeri*. This inhibition decreased after three cycles (72 h) of biological treatment, to 30 ± 4% and 26 ± 7% in the assays without CNM and with CNT@2%Fe, respectively (Table 5). These values reflect a 46% detoxification of the solutions by the anaerobic process. The luminescence inhibition still measured after the anaerobic treatment may be related to the presence of CIP still existing, in the treated solutions, even though at lower amount, as the removal extent in these assays was 86–88% (Table 4). Alternatively, it may be linked to the possible by-products formed during the degradation cycles, which seems most probable considering the high CIP removal verified in the biological assays [73].

The possible contribution of CNM to the toxicity of the treated solution may not be neglected as the treated solutions may contain traces of small amorphous materials from CNM or even impurities that remained in the solutions after removing the CNM [16,74]. In this sense, the toxic extent of the anaerobic medium, previously incubated with CNM under anaerobic conditions, was also assessed. The INH (%) obtained for the medium incubated with CNT and CNT@2%Fe, was 4.7 ± 0.7% and 18.1 ± 1.7, respectively (Table 5). According to Mendonça et al. [75], the toxicological effects of CNM used in this study are considered negligible, since the luminescence variations could be associated to the adaption of the microorganisms to the presence of the pollutant [75,76]. Moreover, the luminescence inhibition caused by the medium itself, without CNM, was 4.9 ± 0.9%, a value similar to that obtained with the medium incubated with CNT, which confirms that CNT do not contributed for the toxicity obtained with treated solutions. It is important to state that the amount of CNM needed to act as RM is very low, only 0.1 g L^−1^, so the amount of possible amorphous CNM released to the medium will also be very low, another advantage of using these materials.

Some authors have reported that the iron in CNM have toxic effects, and that the toxic mechanisms are related to the fact that iron can be leachate from the CNT during the incubation time, and due to the high affinity of iron oxides to the cells membrane, generating reactive oxygen species, which could lead to cells death [77,78]. However, because the efficiency of the material was maintained during the cycles, the material probably maintained the initial structure. Nevertheless, due to its magnetic properties, CNT@2%Fe can be easily removed from the treated water by applying a magnetic field. Therefore, it is not expected that the solutions treated in the presence of this CNM will constitute a toxicity problem when discharged.

## 3. Materials and Methods

### 3.1. Chemicals

CIP was obtained from Sigma-Aldrich, at the purity of 98%. A stock solution of CIP was prepared in deionized water at a concentration of 0.15 mmol L^−1^. Due to the low solubility of CIP, a few drops of hydrochloric acid (2 mol L^−1^) were added, under constant magnetic stirring. CIP has a water solubility of 30 g L^−1^ (0.091 mol L^−1^) at 20 °C and its solubility is enhanced when it is in the ionic form as explained in sub-Section 3.2. Sodium sulfide (Na_2_S.9H_2_O) was purchased from Fluka. Fe(NO_3_)_3_, used for the CNT impregnation with 2% Fe (wt.%), was purchased from Sigma-Aldrich. Formic acid and acetonitrile (ACN) for High Performance Liquid Chromatography (HPLC) analysis were purchased from Merk and Fluka, respectively, at the highest analytic grade purity commercially available (98%). All the reagents used for the preparation of the anaerobic basal medium [79]. were purchased from Sigma-Aldrich. ZnSO_4_.7H_2_O, obtained from ACS, Panreac, was used in the toxicity assessment.

### 3.2. Carbon Nanomaterials

Commercial multiwalled CNT (NC3100TM, Nanocyl SA., Sambreville, Belgium), with 1.5 μm average length, 9.5 nm average diameter and more than 95% carbon purity (according to the supplier’s technical data sheet) were used in the experiments. In order to obtain CNT with N-groups incorporated (sample CNT_N), commercial CNT were mixed with 0.26 g of N using melamine as nitrogen precursor, and the mixture was ball milled in a closed flask without any gas flow in a Retsch MM200 equipment, during 4 h at a constant vibration frequency of 15 vibrations s^−1^. Following, the CNT_N were subjected to a thermal treatment under N_2_ flow (100 cm^3^ min^−1^), until 600 °C and kept at this temperature during 1 h, as previously reported by Soares et al. [51]. A CNT sample with high amount of oxygen-containing surface groups, and consequently strong acid character (sample CNT_HNO_3_) was also prepared through oxidative treatment of the commercial CNT with 7 mmol L^−1^ of HNO_3_, in liquid phase, at boiling temperature, during 3 h as described by Gonçalves et al. [35]. Subsequently, CNT were washed with distilled water to neutral pH, and dried in an oven at 110 °C for 24 h.

Commercial CNT were also impregnated with a metal phase (2%Fe), thus originating a magnetic carbon-based nanocomposite (CNT@2%Fe). CNT were supplemented with 2% Fe by incipient wetness impregnation from aqueous solution of the corresponding metal salt (Fe(NO_3_)_3_). Then, samples were dried at 100 °C for 24 h and placed under nitrogen flow at 400 °C for 1 h, and reduced at 400 °C in hydrogen flow for 3 h [30,80].

Textural properties of CNM, such as the specific surface area (*S*_BET_) and total pore volume (*Vp*), were analyzed, as well as the pH at point of zero charge (pH_PZC_). Elemental analysis and oxygen analysis were also carried out.

Scanning Electron Microscopy with Energy Dispersive Spectroscopy (SEM/EDS) analyses were obtained by using a Schottky scanning electron microscope of high resolution with microanalysis with X-rays and analysis of patterns of diffuse scattering electrons: Quanta 400FEG ESEM/EDAX Genesis X4M.

### 3.3. Effect of CNM on the Chemical Reduction of CIP

In this assay, pristine or tailored CNT (CNT_N and CNT_HNO_3_) were tested to verify which was the best catalyst to be used posteriorly as RM on the biological assays. Chemical reduction of CIP was performed in 70 mL serum bottles with 25 mL basal medium, buffered at a pH of 7.3 ± 0.2 with NaHCO_3_ (2.5 g L^−1^), as described by Angelidaki et al. [79]. This pH was selected based on the fact that it is the required for the biological assays. CNM were added to the vials at a concentration of 0.1 g L^−1^. The bottles were sealed with butyl rubber stoppers and aluminum caps and flushed with N_2_:CO_2_ (80:20% *v*/*v*). Na_2_S was added as reducing agent from a partially neutralized stock solution (0.1 mol L^−1^ Na_2_S), to obtain an initial concentration of 1 mmol L^−1^. The flasks were incubated overnight (14 h) at 37 °C in a rotary shaker (120 rpm), after which CIP was added at a concentration of 1 mmol L^−1^. This relatively high CIP concentration was chosen to facilitate ascertaining whether it is susceptible to being reduced under anaerobic conditions. Further, controls without Na_2_S were prepared. CIP concentration was analyzed by HPLC over 96 h of incubation.

### 3.4. Anaerobic Removal of CIP Assisted by CNM and Characterization of the Inoculum Sludge

Anaerobic assays were performed in 200 mL serum bottles containing 100 mL basal medium, supplemented with micro and macro nutrients, salts, and vitamins, as described by Angelidaki et al. [79]. Anaerobic medium was buffered at a pH of 7.3 ± 0.2 with NaHCO_3_ (2.5 g L^−1^). The anaerobic granular sludge (GS) used as inoculum, was originated from a brewery plant, collected and transported in a closed container of 25 L and preserved at 4 °C, under anaerobic conditions (by flushing the headspace with nitrogen). GS was used at a final volatile solids (VS) concentration of 3 g L^−1^. The bottles were supplemented with the CNM (CNT or CNT@2%Fe) at a concentration of 0.1 g L^−1^. CNT were selected to be tested in biological assays based on the results obtained from the screening of CIP chemical reduction. Because conferring a magnetic character to CNT is beneficial to facilitate their removal after the process, CNT impregnated with 2% were prepared and also used in the biological assays. Bottles were sealed with butyl rubber stoppers and aluminum caps, flushed with N_2_/CO_2_ (80:20% *v*/*v*) and incubated overnight (14 h) at 37 °C and 120 rpm, for the consumption of any residual substrate. After that pre-incubation period, CIP was added at a concentration of 0.015 mmol L^−1^, as well as ethanol (as primary electron donor) at the concentration of 30 mmol L^−1^, from a stock solution of 3 mol L^−1^. Control assays without CNM were also prepared, as well as blank assays without ethanol. Abiotic controls, set up with CNM and ethanol but without sludge, were also included. All the assays were made in triplicate and were incubated at 37 °C, 120 rpm. To verify the reusability and the evolution of the catalytic properties of the materials, CNT@2%Fe, two additional cycles of 24 h were performed in the bottles containing this CNM (Figure S3). For that, after each 24 h, the bottles were flushed with N_2_/CO_2_ (80:20% *v*/*v*) to remove the methane produced, and ethanol (30 mmol L^−1^) and CIP (0.015 mmol L^−1^) were added again to each condition. Furthermore, biological controls without CIP, in the presence and absence of CNM were prepared to better understand the effect of CIP on the acetogenic bacteria which consume ethanol and on methanogenic archaea, producing methane. CIP, ethanol, acetate and methane concentrations were monitored by HPLC and Gas Chromatography (GC), over the time in the experiments.

In order to assess the microbial composition of the anaerobic sludge, aliquots of the inoculum sludge were taken in duplicate and preserved with RNA later (Sigma-Aldrich) at −20 °C. RNA extraction, 16S rRNA sequencing and bioinformatics analysis were performed as described by Salvador et al. [81], with minor changes namely the utilization of primer Uni1492r [82] in the cDNA synthesis step, and the universal primer set 515F/806R [83] targeting the prokaryotic 16S rRNA gene, in sequencing amplification by Illumina MiSeq. FASTQ files containing the 16S rRNA sequences, were deposited in the European Nucleotide Archive (ENA), under the study accession number PRJEB43083.

### 3.5. Analytical Methods

Textural properties such as total specific surface area (*S*_BET_) and total pore volume (*Vp*) at P/P0 = 0.95 were analyzed by N_2_ adsorption isotherms at −196 °C using a Quantachrome NOVA 4200e multi-station equipment, where the samples were previously degassed in vacuum for 3 h at 150 °C. *S*_BET_ was calculated from the nitrogen adsorption data in the relative pressure range of 0.05–0.3 [84]. Thermogravimetric analysis was performed in a NetzschSTA 409 PC Luxx^®^. The analyses were carried out under a helium flow, at a heating rate of 10 °C min^−1^ from 50 to 900 °C, using two isothermal steps at 900 °C: 7 min under helium flow and 13 min under air flow.

The pH at point of zero charge (pH_PZC_) was also determined for each CNM. For that propose, 50 cm^3^ of 0.01 M NaCl solution was placed in closed Erlenmeyer flask and the pH was adjusted to a value between 2 and 10 with the solutions of 0.1 M HCl or 0.1 M NaOH. Then, 0.15 g of each CNM was added and the final pH measured after 24 h under agitation at room temperature. The pH_PZC_ was obtained by the intersection of the curve pH_final_ vs. pH_initial_ with the line pH_initial_ = pH_final_ [85].

Each element (CHNS) was determined on a vario MICRO cube analyzer from Elemental GmbH in CHNS mode, by combustion of the sample at 1050 °C and calculated by the mean of three independent measurements, using a per-day calibration with a standard compound. Oxygen composition was determined a rapid OXY cube analyzer from Elemental GmbH, by pyrolysis of the sample at 1450 °C and calculated by the mean of three independent measurements, using a per-day calibration with a standard compound [37].

The vs. were determined gravimetrically as described in Standard Methods [86].

Removal of CIP was assessed by HPLC analysis, based on the disappearance of its corresponding peak at retention time of 12.5 min (Figure S4). The analyses were performed as previously reported by Silva et al. [73]. An Ultra HPLC (Nexera XZ, Shimadzu, Japan) equipped with a Diode Array Detector (SPD-M20A), an autosampler (SIL-30AC), degassing unit (DGU-20A5R), LC-30AD solvent delivery unit, a Labsolutions software and a RP-18 endcapped Purospher Star column (250 × 4 mm, 5 μM particle size, from MERK, Germany) were used. The mobile phase was composed by 0.1% formic acid solution (solution A) and ACN (solution B). Prior to analysis, samples were centrifuged (10 min at 10,000 rpm) and filtered (Whatman SPARTAN syringe filters, regenerated cellulose, 0.2 μm pore size). The compounds were eluted at a flow rate of 0.8 mL min^−1^ at 40 °C, with the following gradient: increase of ACN from 5 to 15%, over 6 min, followed by an isocratic step during 12 min, then from 15% to 40% of ACN during 12 min and 40% was then maintained for 10 min. A calibration curve at increasing CIP concentrations from 0.0002 to 0.03 mmol L^−1^ was made.

The percentage of CIP removal (*PR*) was calculated according to Equation (4):

(4) $$PR\;\left(\%\right)=\frac{\left(C_{0}-C_{t}\right)}{C_{0}}\;\times 100$$

where *C*_0_ is the initial CIP concentration and *C_t_* the CIP concentration at time t.

First-order reduction rate constants were calculated in OriginPro 6.1. software, applying Equation (5):

(5) $$C_{t}=C_{i}+Ae^{-t/k}$$

where *C_t_* is defined in equation 1, *C_i_* is the offset, a value closed to the asymptotic of the *Y* variable (*C*) for larger time (*t*) values and k is the first-order rate constant (d^−1^).

Ethanol and acetate were also monitored by HPLC (Jasco, Tokyo, Japan), with a RI and UV detector (at 210 nm), respectively, using a Rezex ROA Organic Acid H^+^ (8%), (300 mm × 7.8 mm) column. The elution was made at 60 °C using sulfuric acid (5 mmol L^−1^) as mobile phase, at a flow rate of 0.6 mL min^−1^.

The concentration of CH_4_ produced in each bottle, over each degradation cycle, was assessed by gas chromatography (GC), using a Shimadzu GC-2014 gas chromatograph fitted with Porapak Q 80/100 mesh, packed stainless-steel column (2 m × 1/8 inch, 2 mm) and a flame ionization detector (FID). Nitrogen was the carrier gas at a flow rate of 30 mL min^−1^ and the column, injection port and detector temperatures were respectively 35, 110, and 220 °C. Headspace gas was sampled by a 500 μL pressure-lock syringe (Hamilton). The values of CH_4_ production were corrected for the standard temperature and pressure conditions (STP). A standard sample composed of 40% of CH_4_ was injected firstly, followed by samples injection.

### 3.6. Statistical analysis

Statistical significance of the differences in the biological CIP reduction rates and methane production rates obtained after each degradation cycle, was evaluated using single factor analysis of variances (ANOVA). Statistical significance was established at the *p* < 0.05 level.

### 3.7. Toxicity Assessment with Vibrio fischeri

Toxicity assays were performed with *V. fischeri* strain NRRL-B-1117, purchased as freeze-dried reagent, BioFix^®^ *Lumi*, from Macherey-Nagel (Düren, Germany) and grown under aerobic conditions, as described in the international standard ISO 11348-1 “Water quality–Determination of the inhibition effect of water samples on the light emission of *Vibrio fischeri* (Luminescent bacteria test)” method, using freshly prepared bacteria [87].

Toxicity was assessed for samples collected at the end of the third cycle (72 h of incubation) in the bottles containing CNT@2%Fe, since this is the condition that better represents the contribution of all the mechanisms for CIP removal. Samples were centrifuged (10 min at 10,000 rpm) and filtered (Whatman SPARTAN syringe filters, regenerated cellulose, 0.2 μm pore size) prior to the toxicity assay. CIP solution (0.015 mmol L^−1^) and anaerobic medium were also tested. Moreover, solutions containing 0.1 g L^−1^ of CNT and CNT@2%Fe were prepared in anaerobic medium and placed at 37 °C and 120 rpm, during 72 h. After that period, samples were collected and centrifuged, and the toxicity of the supernatant was evaluated. Evaluation of pristine CNT, besides of CNT@2%Fe, allows assessing whether iron impregnation makes the material more toxic or not. Negative controls were prepared with the bacterial suspension and a solution of 2% NaCl. Zinc sulfate heptahydrate at a concentration of 19.34 mg L^−1^ was used as positive control [88]. The salinity of all the samples and solutions was adjusted to 2% NaCl. The samples pH was adjusted to values between 6 and 9 with hydrochloric acid or sodium hydroxide. Oxygen concentration was higher than 3 mg L^−1^, and turbidity was avoided by samples centrifugation and filtration.

Toxicity evaluation was performed according to the standard ISO 11348-1 and 11348-3, using a microplate reader (Biotek^®^ Cytation3, Fisher Scientific, Korea) in kinetic mode to evaluate the bacteria luminescence changes when exposed to potentially toxic substances [87,89]. For that propose, a 96 well optical Btm Plt polymer base Blk plate, from Nalge Nunc™ International, was used, where each sample (100 μL) was mixed with the bacteria test suspension (100 μL), according to the ISO 11348-3.

Luminescence inhibition (*INH %*) was calculated after 30 min [87,88,89], according to Equation (6):

(6) $$INH\;\left(\%\right)=100-\frac{IT_{t}}{KF\times IT_{0}}\times 100$$

with

(7) $$KF=\frac{IC_{t}}{IC_{0}}$$

where *IT_t_* is luminescence intensity of the sample after the contact time (30 min), *IT*_0_ is the luminescence intensity at the beginning of the assay (time 0), *KF* is the correction factor and characterizes the natural loss of luminescence of the negative control, *IC_t_* is the luminescence intensity of the control after the contact time and *IC*_0_ is the initial luminescence intensity of the negative control. The luminescence signal was recorded in relative light units (RLU s^−1^).

## 4. Conclusions

In this work, high extent of CIP removal was obtained by applying an anaerobic treatment supplemented with CNM. CIP removal was attained either by adsorption on GS and CNM, or by a combined effect of sorption and biological removal in anaerobic conditions. The presence of CNM increased the rates of CIP removal ≈ 1.5-fold, highlighting the potential of these nanomaterials to improve the efficiency of the processes. The anaerobic treatment applied, both in the absence and in the presence of the CNM, caused a significant decrease in the toxicity (around 50%), with all the treated solutions being considered slightly toxic, while the initial CIP solution was toxic. Therefore, the use of CNM may be advantageous to increase the removal efficiency of this pharmaceutical compound, and still water detoxification. The application of magnetic nanomaterials, like CNT@2%Fe, facilitates their separation and removal from the process after treatment, by applying a magnetic field, which is an advantage relatively to soluble and other insoluble materials. Furthermore, this magnetic CNM, maintained its good catalytic properties over three treatment cycles, demonstrating its recycling and reusability in anaerobic systems.

## Figures and Tables

**Table 1:** Surface, textural, and elemental analysis of the different carbon nanotubes (CNT).

Sample	CNT	CNT_N	CNT_HNO_3_	CNT@2%Fe
S_BET_ (m^2^ g^−1^)	201	225	223	196
Vp (cm^3^ g^−1^)	0.416	0.503	0.448	0.440
pH_PZC_ (±0.2)	6.6	6.7	2.2	6.5
N (%) *	0.00	1.69	0.00	n.d.
C (%) *	99.8	96.4	98.0	n.d.
H (%) *	0.11	0.18	0.19	n.d.
S (%) *	0.00	0.00	0.15	n.d.
O (%) *	0.06	0.39	1.25	n.d.

* Determined by elemental analysis. n.d.—Not determined. CNT = carbon nanotubes; CNT_N = CNT with N-groups incorporated; CNT_HNO_3_ = oxidized with HNO_3_; CNT@2%Fe = CNT impregnated with 2% Fe.

**Table 2:** Chemical reduction of ciprofloxacin (CIP) (1 mmol L^−1^) by Na_2_S (1 mmol L^−1^) in the absence and presence of the different CNM.

Sample	Removal (%)	Rate (mmol L^−1^ d^−1^)
No CNM	0	0
CNT	42.6 ± 5.0	0.082 ± 0.001
CNT_N	30.1 ± 8.6	0.063 ± 0.001
CNT_HNO_3_	0	0

CNT = carbon nanotubes; CNT_N = CNT with N-groups incorporated; CNT_HNO_3_ = oxidized with HNO_3_.

**Table 3:** Percentage of CIP removal (%) and rate (d^−1^) in the anaerobic assays, performed in the absence and presence of CNM. Blank controls without ethanol, as well as abiotic assays without granular sludge are also presented.

		Cycle 1		Cycle 2		Cycle 3	
Condition		CIP Removal (%)	Rate (d^−1^)	CIP Removal (%)	Rate (d^−1^)	CIP Removal (%)	Rate (d^−1^)
Biotic assays	GS + CIP + E	95 ± 1.0	1.67 ± 0.4	89 ± 3.3	1.39 ± 0.4	86 ± 2.2	1.41 ± 0.2
	GS + CIP + E + CNT	97 ± 0.7	2.24 ± 0.3	n.a.	n.a.	n.a.	n.a.
	GS + CIP + E + CNT@2%Fe	94 ± 0.5	2.55 ± 0.1	90 ± 8.6	1.49 ± 0.2	88 ± 4.1	1.54 ± 0.3
Blank assays	GS + CIP	90 ± 0.1	1.16 ± 0.1	79 ± 2.3	0.92 ± 0.2	68 ± 5.7	1.07 ± 0.1
	GS + CIP + CNT	94 ± 0.1	2.7 ± 0.1	n.a.	n.a.	n.a.	n.a.
	GS + CIP + CNT@2%Fe	89 ± 0.2	2.4 ± 0.1	84 ± 2.6	1.7 ± 0.6	78 ± 0.8	0.99 ± 0.2
Abiotic assays	CIP + E +CNT	98 ± 0.5	1.67 ± 0.4	n.a.	n.a.	n.a.	n.a.
	CIP + E + CNT@2%Fe	99 ± 0.4	1.32 ± 0.6	79 ± 8.3	0.3 ± 0.1	29 ± 3.2	0.13 ± 0.1

n.a.—Not applicable. GS = granular sludge; CIP = ciprofloxacin; E = ethanol; CNT = carbon nanotubes; CNT@2%Fe = carbon nanotubes impregnated with 2% Fe.

**Table 4:** Rates of ethanol consumption and methane production, over 3 cycles of biological removal of CIP in the presence of CNM.

	Condition	Ethanol Consumption Rate (mmol L^−1^h^−1^)			Methane Production Rate (mmol L^−1^h^−1^)		
		Cycle 1	Cycle 2	Cycle 3	Cycle 1	Cycle 2	Cycle 3
Biotic assays	GS + E	3.24 ± 0.62	3.76 ± 1.16	3.26 ± 0.45	2.58 ± 0.05	2.78 ± 0.06	3.03 ± 0.03
	GS + E + CNT	3.66 ± 0.50	n.a.	n.a.	2.62 ± 0.04	n.a.	n.a.
	GS + E + CNT@2%Fe	3.57 ± 0.34	3.72 ± 1.08	3.32 ± 0.51	2.23 ± 0.20	2.84 ± 0.03	3.07 ± 0.03
	GS + CIP + E	3.41 ± 0.46	3.29 ± 0.63	3.21 ± 0.24	2.61 ± 0.03	2.89 ± 0.03	3.00 ± 0.06
	GS + CIP + E + CNT	3.08 ± 0.30	n.a.	n.a.	2.51 ± 0.08	n.a.	n.a.
	GS+ CIP+ E + CNT@2%Fe	3.39 ± 0.47	3.23 ± 0.67	3.27 ± 0.53	2.31 ± 0.20	2.86 ± 0.03	2.92 ± 0.03

n.a.—Not applicable. GS = granular sludge; CIP = ciprofloxacin; E = ethanol; CNT = carbon nanotubes; CNT@2%Fe = carbon nanotubes impregnated with 2% Fe.

**Table 5:** Luminescence inhibition (INH) of *V. fischeri* in all the tested samples, after 30 min of exposure.

Samples		INH (%)
CIP solution (0.015 mmol L^−1^)		56 ± 10
GS + CIP + E (Treatment of 72 h)		30 ± 4
GS + CIP + E + CNT@2%Fe (Treatment of 72 h)		26 ± 7
Positive control (ZnSO_4_.7H_2_O)		83 ± 8
Anaerobic medium		4.9 ± 0.9
Medium after incubation with 0.1 g L^−1^ of CNM	CNT	4.7 ± 0.7
	CNT@2%Fe	18.1 ± 1.7

## Data Availability

The data that support the findings of this study are available from the corresponding author upon reasonable request.

## Supplementary Materials

The following are available online at https://www.mdpi.com/1422-0067/22/6/2932/s1.
