# Supplementary material for: Detoxification of Ciprofloxacin in an Anaerobic Bioprocess Supplemented with Magnetic Carbon Nanotubes: Contribution of Adsorption and Biodegradation Mechanisms
Source: Int J Mol Sci. 2021 Mar 13;22(6):2932. doi: 10.3390/ijms22062932 (PMC7999377; doi:10.3390/ijms22062932)
Supplement: Supplementary file 1 [file ijms-22-02932-s001.pdf]

International Journal of *Molecular Sciences*

Supplementary Material

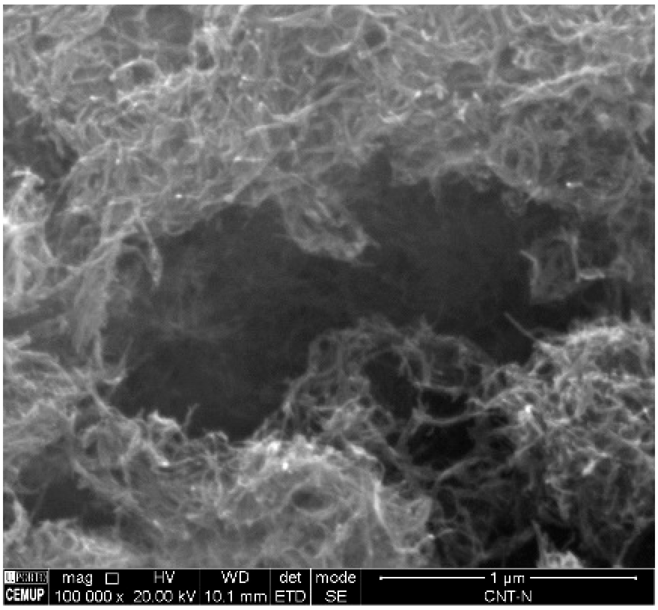

Figure S1. SEM images of CNT_N after ball milling treatment.

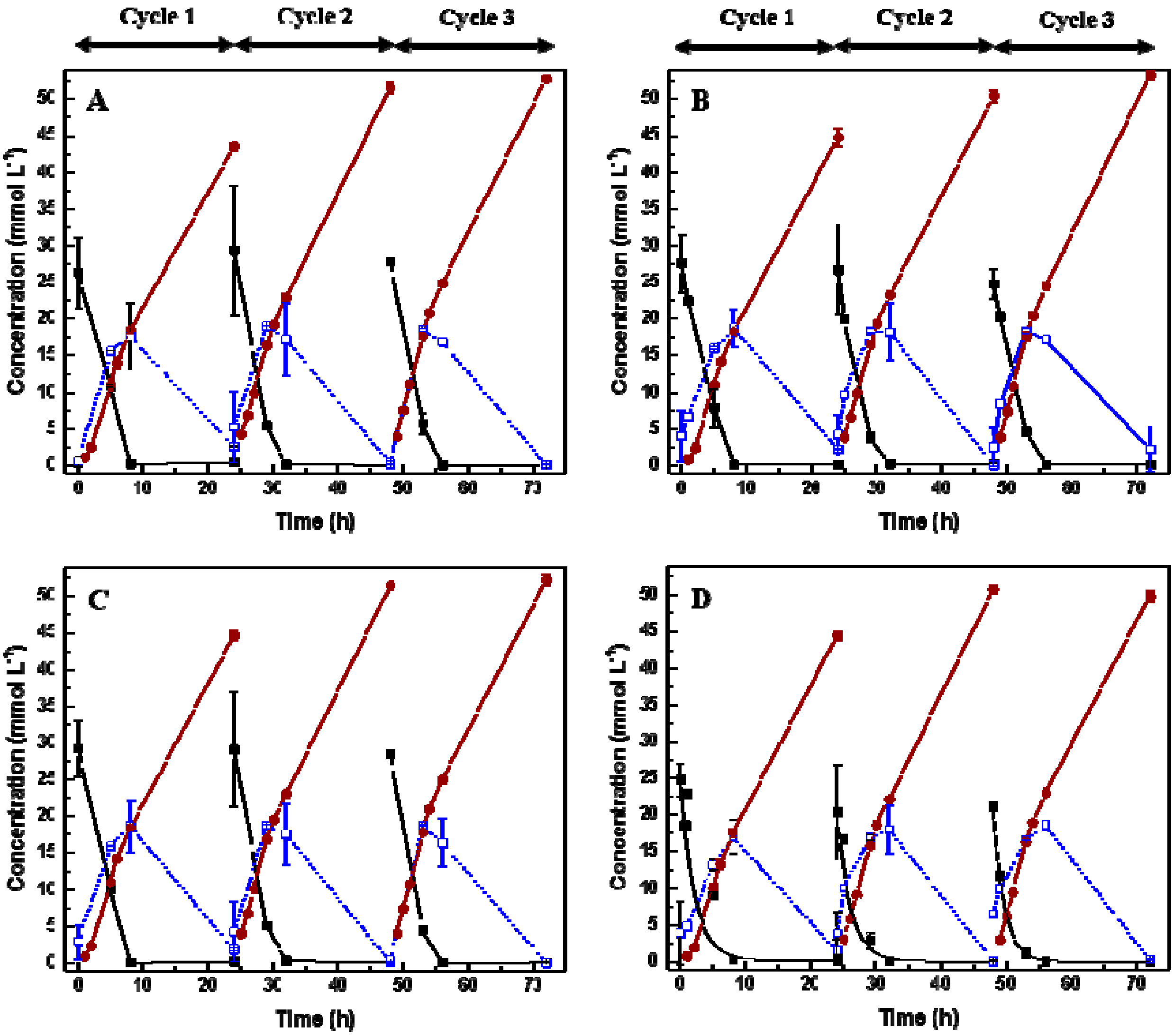

Figure S2. Ethanol conversion in the anaerobic assays: (A) GS+E, (B) GS+CIP+E, (C) GS+E+CNT@2%Fe and (D) GS+CIP+E+CNT@2%Fe, over 3 cycles of CIP removal: ethanol (■), acetate (□) and methane (●) concentrations.

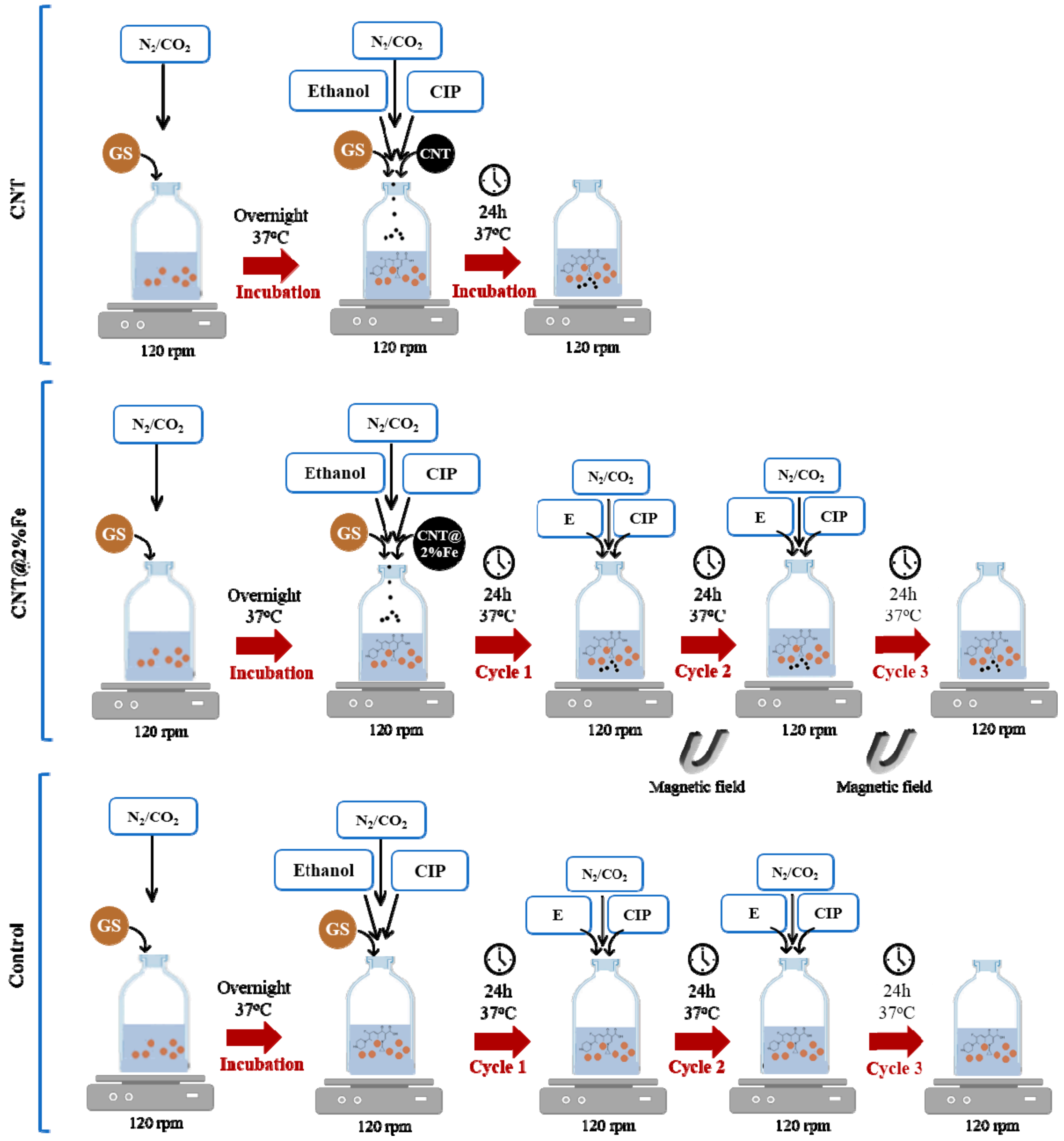

Figure S3. Experimental setup of the biological assays in the presence and absence of CNM (GS + CIP + E + CNT, GS + CIP + E + CNT@2%Fe and GS + CIP + E). For blank and abiotic controls, substrate and GS was not added, respectively.

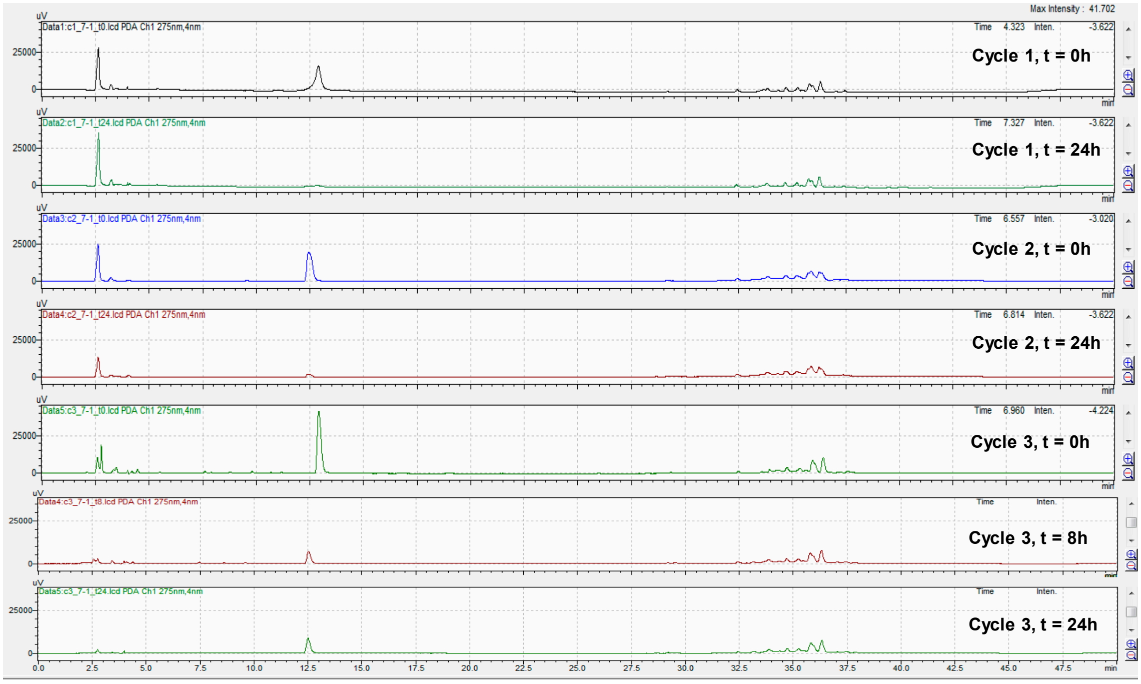

Figure S4. HPLC chromatograms of the biological removal of CIP, in the presence of CNT@2%Fe (GS+CIP+E+CNT@2%Fe), at the beginning (t=0h) and after 24 h of reaction in the three cycles of CIP addition. CIP was detected at the RT= 12.5 min and at 275 nm.

Table S1. Removal of CIP (1 mmol L^{-1}) in the absence and presence of the different CNM in the reactional medium without Na_2S

**Sample**	**Removal (%)**
No CNM	0
CNT	3.84
CNT_N	3.29
CNT_HNO_3	3.18

Table S2. Ethanol conversion in the anaerobic assays, over 3 cycles of CIP removal: ethanol, acetate and methane concentrations (mmol L^{-1}) detected at the end of each cycle

	Condition	Ethanol (mmol L^{-1})			Acetate (mmol L^{-1})			Methane (mmol L^{-1})		
		Cycle 1	Cycle 2	Cycle 3	Cycle 1	Cycle 2	Cycle 3	Cycle 1	Cycle 2	Cycle 3
Biotic assays	GS + E	0.43 ± 0.4	0.05 ± 0.04	0.03 ± 0.04	2.56 ± 0.03	0.34 ± 0.2	0.10 ± 0.1	43.5 ± 0.4	51.5 ± 0.7	50.9 ± 0.1
	GS + E+ CNT	0.17 ± 0.1	n.a.	n.a.	1.75 ± 0.23	n.a.	n.a.	44.7 ± 0.7	n.a.	n.a.
	GS + E + CNT@2%Fe	0.19 ± 0.01	0.09 ± 0.1	0.09 ± 0.02	5.14 ± 0.9	0.10 ± 0.2	0 ± 0	48.9 ± 1.9	51.5 ± 0.1	50.3 ± 0.7
	GS + CIP + E	0.1 ± 0	0.18 ± 0.1	0.08	2.21 ± 0.1	0 ± 0	2.23 ± 3.2	44.8 ± 1.3	50.4 ± 0.8	51.3 ± 0.5
	GS + CIP + E + CNT	0.12 ± 0.01	n.a.	n.a.	1.40 ± 1.6	n.a.	n.a.	44.4 ± 0.6	n.a.	n.a.
	GS + CIP + E + CNT@2%Fe	0.89 ± 1.3	0.03 ± 0.1	0	3.7 ± 2.3	0 ± 0	0.27 ± 0.03	48.5 ± 0.2	50.7 ± 0.6	47.9 ± 0.8
Blank assays	GS + CIP	n.a.	n.a.	n.a.	n.a.	n.a.	n.a.	0 ± 0.03	0 ± 0.02	0 ± 0.01
	GS+ CIP + CNT	n.a.	n.a.	n.a.	n.a.	n.a.	n.a.	0 ± 0.03	n.a.	n.a.
	GS+ CIP + CNT@2%Fe	n.a.	n.a.	n.a.	n.a.	n.a.	n.a.	0 ± 0.04	0 ± 0.04	0 ± 0.04
Abiotic assays	CIP + E + CNT	28.23 ± 7.6	n.a.	n.a.	n.a.	n.a.	n.a.	n.a.	n.a.	n.a.
	CIP + E + CNT@2%Fe	30 ± 1.0	39.4 ± 10.3	76.8	n.a.	n.a.	n.a.	n.a.	n.a.	n.a.

n.a. – Not - applicable

Table S3. Taxonomic characterization of anaerobic inoculum sludge, based on 16S rRNA gene sequencing. The relative abundance of each taxon (given in percentage) is the average and standard deviation for duplicate samples

Kingdom	Phylum	Class	Family	Genus	Relative abundance (%)		
Archaea	*Euryarchaeota*	*Methanobacteria*	*Methanobacteriaceae*	*Methanobacterium*	8.9870	±	3.2533
Archaea	*Euryarchaeota*	*Methanobacteria*	*Methanobacteriaceae*	*Methanobrevibacter*	0.0034	±	0.0048
Archaea	*Euryarchaeota*	*Methanomicrobia*	*Methanoregulaceae*	*Methanolinea*	9.0534	±	0.6312
Archaea	*Euryarchaeota*	*Methanomicrobia*	*Methanospirillaceae*	*Methanospirillum*	0.0736	±	0.0244
Archaea	*Euryarchaeota*	*Methanomicrobia*	*Methanosaetaceae*	*Methanosaeta*	19.9613	±	0.9552
Archaea	*Euryarchaeota*	*Methanomicrobia*	*Methanosarcinaceae*	*Methanomethylovorans*	0.0258	±	0.0018
Archaea	*Euryarchaeota*	*Methanomicrobia*	*Unclassified*	*Unclassified*	0.0236	±	0.0047
Archaea	*Euryarchaeota*	*Thermoplasmata*	*Unclassified*	*Unclassified*	0.0956	±	0.0141
Archaea	*Crenarchaeota*	*Unclassified*	*Unclassified*	*Unclassified*	0.0616	±	0.0212
Archaea	*Unclassified*	*Unclassified*	*Unclassified*	*Unclassified*	0.7637	±	0.0725
Bacteria	*Acidobacteria*	*Acidobacteriia*	*Unclassified*	*Unclassified*	0.0654	±	0.0127
Bacteria	*Actinobacteria*	*Actinobacteria*	*Unclassified*	*Unclassified*	0.0034	±	0.0048
Bacteria	*Actinobacteria*	*Actinobacteria*	*Unclassified*	*Unclassified*	0.0167	±	0.0077
Bacteria	*Actinobacteria*	*Thermoleophilia*	*Solirubrobacteraceae*	*Solirubrobacter*	0.0620	±	0.0079
Bacteria	*Actinobacteria*	*Unclassified*	*Unclassified*	*Unclassified*	0.0034	±	0.0048
Bacteria	*Bacteroidetes*	*Bacteroidia*	*Marinilabiliaceae*	*Anaerophaga*	0.0389	±	0.0040
Bacteria	*Bacteroidetes*	*Bacteroidia*	*Bacteroidaceae*	*Bacteroides*	0.5949	±	0.0290
Bacteria	*Bacteroidetes*	*Cytophagia*	*Cytophagaceae*	*Cytophaga*	0.1722	±	0.0203
Bacteria	*candidate division NC10*	*Unclassified*	*Unclassified*	*Unclassified*	0.0189	±	0.0045
Bacteria	*Chlorobi*	*Chlorobia*	*Chlorobiaceae*	*Chlorobium*	0.0024	±	0.0001

Table S3.1. Continued. Taxonomic characterization of anaerobic inoculum sludge, based on 16S rRNA gene sequencing. The relative abundance of each taxon (given in percentage) is the average and standard deviation for duplicate samples

Kingdom	Phylum	Class	Family	Genus	Relative abundance (%)		
Bacteria	*Chloroflexi*	*Anaerolineae*	*Anaerolineaceae*	*Levilinea*	0.0396	±	0.0109
Bacteria	*Chloroflexi*	*Anaerolineae*	*Anaerolineaceae*	*Pelolinea*	0.0035	±	0.0015

Bacteria	*Chloroflexi*	*Anaerolineae*	*Anaerolineaceae*	*Anaerolinea*	0.9173	±	0.2107
Bacteria	*Chloroflexi*	*Anaerolineae*	*Anaerolineaceae*	*Bellilinea*	0.0290	±	0.0132
Bacteria	*Chloroflexi*	*Anaerolineae*	*Anaerolineaceae*	*Flexilinea*	0.0174	±	0.0073
Bacteria	*Chloroflexi*	*Anaerolineae*	*Anaerolineaceae*	*Leptolinea*	0.0141	±	0.0009
Bacteria	*Chloroflexi*	*Anaerolineae*	*Anaerolineaceae*	*Longilinea*	0.3884	±	0.0564
Bacteria	*Chloroflexi*	*Anaerolineae*	*Unclassified*	*Unclassified*	0.1284	±	0.0288
Bacteria	*Chloroflexi*	*Caldilineae*	*Caldilineaceae*	*Caldilinea*	0.0107	±	0.0056
Bacteria	*Chloroflexi*	*Caldilineae*	*Unclassified*	*Unclassified*	0.0412	±	0.0008
Bacteria	*Chloroflexi*	*Chloroflexia*	*Unclassified*	*Unclassified*	0.0269	±	0.0034
Bacteria	*Chloroflexi*	*Dehalococcoidia*	*Dehalococcoidaceae*	*Dehalococcoides*	0.0167	±	0.0077
Bacteria	*Chloroflexi*	*Dehalococcoidia*	*Unclassified*	*Dehalogenimonas*	0.0081	±	0.0045
Bacteria	*Chloroflexi*	*Unclassified*	*Unclassified*	*Unclassified*	0.1965	±	0.0314
Bacteria	*Deferribacteres*	*Deferribacteres*	*Deferribacteraceae*	*Deferribacter*	0.0036	±	0.0019
Bacteria	*Deferribacteres*	*Unclassified*	*Unclassified*	*Unclassified*	0.0095	±	0.0039
Bacteria	*Firmicutes*	*Unclassified*	*Unclassified*	*Unclassified*	0.0743	±	0.0095
Bacteria	*Firmicutes*	*Clostridia*	*Clostridiaceae*	*Clostridium*	0.0223	±	0.0003
Bacteria	*Firmicutes*	*Clostridia*	*Christensenellaceae*	*Christensenella*	0.0140	±	0.0025
Bacteria	*Firmicutes*	*Clostridia*	*Lachnospiraceae*	*Unclassified*	0.0011	±	0.0016
Bacteria	*Firmicutes*	*Clostridia*	*Peptococcaceae*	*Dehalobacter*	0.0057	±	0.0046
Bacteria	*Firmicutes*	*Clostridia*	*Thermoanaerobacteraceae*	*Thermoanaerobacter*	0.0189	±	0.0045
Bacteria	*Firmicutes*	*Clostridia*	*Unclassified*	*Unclassified*	0.0594	±	0.0180
Bacteria	*Firmicutes*	*Negativicutes*	*Unclassified*	*Unclassified*	0.0037	±	0.0052
Bacteria	*Firmicutes*	*Negativicutes*	*Veillonellaceae*	*Centipeda*	0.0153	±	0.0007

Table S3.2. Continued. Taxonomic characterization of anaerobic inoculum sludge, based on 16S rRNA gene sequencing. The relative abundance of each taxon (given in percentage) is the average and standard deviation for duplicate samples

**Kingdom**	**Phylum**	**Class**	**Family**	**Genus**	**Relative abundance (%)**		
Bacteria	*Ignavibacteriae*	*Ignavibacteria*	*Unclassified*	*Unclassified*	0.0025	±	0.0035

Bacteria	*Nitrospirae*	*Nitrospira*	*Nitrospiraceae*	*Unclassified*	0.2626	±	0.0175
Bacteria	*Planctomycetes*	*Phycisphaerae*	*Unclassified*	*Unclassified*	0.0034	±	0.0048
Bacteria	*Planctomycetes*	*Planctomycetia*	*Unclassified*	*Unclassified*	0.0047	±	0.0003
Bacteria	*Planctomycetes*	*Planctomycetia*	*Unclassified*	*Unclassified*	0.3803	±	0.0245
Bacteria	*Proteobacteria*	*Alphaproteobacteria*	*Brucellaceae*	*Ochrobactrum*	0.0083	±	0.0022
Bacteria	*Proteobacteria*	*Alphaproteobacteria*	*Rhizobiaceae*	*Rhizobium*	0.0023	±	0.0032
Bacteria	*Proteobacteria*	*Betaproteobacteria*	*Burkholderiaceae*	*Ralstonia*	0.0398	±	0.0043
Bacteria	*Proteobacteria*	*Betaproteobacteria*	*Methylophilaceae*	*Methylobacillus*	0.0154	±	0.0026
Bacteria	*Proteobacteria*	*Betaproteobacteria*	*Rhodocyclaceae*	*Propionivibrio*	0.0057	±	0.0046
Bacteria	*Proteobacteria*	*Deltaproteobacteria*	*Unclassified*	*Unclassified*	0.0804	±	0.0148
Bacteria	*Proteobacteria*	*Deltaproteobacteria*	*Desulfovibrionaceae*	*Desulfovibrio*	0.0323	±	0.0202
Bacteria	*Proteobacteria*	*Deltaproteobacteria*	*Desulfomicrobiaceae*	*Desulfomicrobium*	0.4685	±	0.0516
Bacteria	*Proteobacteria*	*Deltaproteobacteria*	*Geobacteraceae*	*Geobacter*	14.3896	±	0.8924
Bacteria	*Proteobacteria*	*Deltaproteobacteria*	*Pelobacteraceae*	*Pelobacter*	0.0392	±	0.0140
Bacteria	*Proteobacteria*	*Deltaproteobacteria*	*Unclassified*	*Unclassified*	0.0023	±	0.0032
Bacteria	*Proteobacteria*	*Deltaproteobacteria*	*Polyangiaceae*	*Chondromyces*	0.0115	±	0.0093
Bacteria	*Proteobacteria*	*Deltaproteobacteria*	*Unclassified*	*Unclassified*	0.6188	±	0.0941
Bacteria	*Proteobacteria*	*Deltaproteobacteria*	*Syntrophaceae*	*Smithella*	0.0908	±	0.0104
Bacteria	*Proteobacteria*	*Deltaproteobacteria*	*Syntrophaceae*	*Desulfomonile*	0.1326	±	0.0070
Bacteria	*Proteobacteria*	*Deltaproteobacteria*	*Syntrophaceae*	*Syntrophus*	0.4445	±	0.0696
Bacteria	*Proteobacteria*	*Deltaproteobacteria*	*Syntrophaceae*	*Unclassified*	0.0633	±	0.0062

Table S3.3. Continued. Taxonomic characterization of anaerobic inoculum sludge, based on 16S rRNA gene sequencing. The relative abundance of each taxon (given in percentage) is the average and standard deviation for duplicate samples

Kingdom	Phylum	Class	Family	Genus	Relative abundance (%)		
Bacteria	*Proteobacteria*	*Deltaproteobacteria*	*Syntrophobacteraceae*	*Syntrophobacter*	15.8377	±	0.8367
Bacteria	*Proteobacteria*	*Deltaproteobacteria*	*Syntrophobacteraceae*	*Unclassified*	0.0035	±	0.0015
Bacteria	*Proteobacteria*	*Deltaproteobacteria*	*Syntrophorhabdaceae*	*Syntrophorhabdus*	0.1084	±	0.0284

Bacteria	*Proteobacteria*	*Deltaproteobacteria*	*Unclassified*	*Unclassified*	0.2745	±	0.0215
Bacteria	*Proteobacteria*	*Epsilonproteobacteria*	*Campylobacteraceae*	*Arcobacter*	0.0023	±	0.0032
Bacteria	*Proteobacteria*	*Epsilonproteobacteria*	*Helicobacteraceae*	*Sulfuricurvum*	0.0326	±	0.0113
Bacteria	*Proteobacteria*	*Gammaproteobacteria*	*Methylococcaceae*	*Methylobacter*	0.0104	±	0.0044
Bacteria	*Proteobacteria*	*Gammaproteobacteria*	*Unclassified*	*Unclassified*	0.0072	±	0.0038
Bacteria	*Proteobacteria*	*Gammaproteobacteria*	*Thiotrichaceae*	*Thiothrix*	0.0012	±	0.0017
Bacteria	*Proteobacteria*	*Gammaproteobacteria*	*Unclassified*	*Unclassified*	0.0252	±	0.0165
Bacteria	*Proteobacteria*	*Unclassified*	*Unclassified*	*Unclassified*	4.1797	±	0.7489
Bacteria	*Spirochaetes*	*Spirochaetia*	*Unclassified*	*Unclassified*	0.0432	±	0.0090
Bacteria	*Spirochaetes*	*Spirochaetia*	*Spirochaetaceae*	*Spirochaeta*	0.0446	±	0.0006
Bacteria	*Spirochaetes*	*Spirochaetia*	*Unclassified*	*Unclassified*	0.1357	±	0.0218
Bacteria	*Spirochaetes*	*Unclassified*	*Unclassified*	*Unclassified*	0.5086	±	0.0076
Bacteria	*Synergistetes*	*Synergistia*	*Unclassified*	*Unclassified*	0.1114	±	0.0300
Bacteria	*Synergistetes*	*Synergistia*	*Synergistaceae*	*Synergistes*	0.0455	±	0.0122
Bacteria	*Synergistetes*	*Synergistia*	*Synergistaceae*	*Unclassified*	0.0284	±	0.0050
Bacteria	*Synergistetes*	*Synergistia*	*Synergistaceae*	*Aminivibrio*	0.0738	±	0.0310
Bacteria	*Synergistetes*	*Synergistia*	*Synergistaceae*	*Aminobacterium*	0.0034	±	0.0048
Bacteria	*Synergistetes*	*Synergistia*	*Synergistaceae*	*Lactivibrio*	0.0011	±	0.0016
Bacteria	*Synergistetes*	*Unclassified*	*Unclassified*	*Unclassified*	0.0071	±	0.0004

Table S3.4. Continued. Taxonomic characterization of anaerobic inoculum sludge, based on 16S rRNA gene sequencing. The relative abundance of each taxon (given in percentage) is the average and standard deviation for duplicate samples

**Kingdom**	**Phylum**	**Class**	**Family**	**Genus**	**Relative abundance (%)**		
Bacteria	*Thermotogae*	*Thermotogae*	*Kosmotogaceae*	*Mesotoga*	0.4912	±	0.1941
Bacteria	*Thermotogae*	*Thermotogae*	*Unclassified*	*Unclassified*	0.0037	±	0.0052
Bacteria	*Unclassified*	*Unclassified*	*Unclassified*	*Unclassified*	18.6068	±	2.7284
Bacteria	*Unclassified*	*Unclassified*	*Unclassified*	*Caldithrix*	0.0047	±	0.0003
Bacteria	*Verrucomicrobia*	*Verrucomicrobiae*	*Unclassified*	*Unclassified*	0.0267	±	0.0100

Bacteria	*Verrucomicrobia*	*Verrucomicrobiae*	*Verrucomicrobiaceae*	*Verrucomicrobium*	0.0069	±	0.0062
